# Hepatopulmonary Fistula in a Colorectal Cancer Patient

**DOI:** 10.1155/2019/1475209

**Published:** 2019-03-04

**Authors:** Maria João Silva, Nuno Almeida Costa, Susana Dias, Maria José Sousa, Maria Fragoso

**Affiliations:** ^1^Medical Oncology Department, Instituto Português de Oncologia do Porto, Francisco Gentil, Oporto, Portugal; ^2^Radiology Department, Instituto Português de Oncologia do Porto, Francisco Gentil, Oporto, Portugal

## Abstract

Cancer patients with liver metastasis may be candidates for liver surgery and local interventional techniques as part of their treatment. Although rare in this setting, hepatopulmonary fistula has been described as a possible complication. The clinical picture may be atypical, and, without specific treatment, it usually has a dismal prognosis. As locoregional treatments (whether interventional or surgical techniques) in liver neoplasms are being more frequently used we need to include this entity as a possible differential diagnosis of local liver treatment complications.

## 1. Introduction

Hepatopulmonary fistula, defined as a communication between the bronchial tree and a hepatic segment, is a rare complication that may occur days to years after surgery [1].

Potential causes of hepatopulmonary fistulas are grouped into five main categories, which have changed prevalence over the years: congenital and hepatic hydatic disease or liver abscess (echinococcic, amoebic, and pyogenic), more frequent in the past, especially in developing countries; biliary tract obstruction secondary to tumors (more frequently biliary tree tumors but also metastatic lesions); blunt or penetrating injury (with or without an expanding hematoma causing obstruction); iatrogenic fistulas, following liver resection, radiofrequency ablation (RFA), external radiation, and thoracic drainage [2]. With the growing use of liver ablating techniques and surgery, iatrogenic causes are becoming gradually more frequent.

Despite technical advances, even in specialized centers with high volume of liver surgery, postoperative morbidity is still a burden with rates around 20-40% [3]. Complex resections are being increasingly performed in high-risk groups, as patients on chemotherapy, with or without antibodies and radiation therapy and with advanced age [4].

## 2. Case Presentation

A 74-year-old man presented to the Emergency Department (ED) on April 2016, after 4 days of fever, productive cough, and abundant “yellow” sputum; he was started on oral levofloxacin on the diagnosis of tracheobronchitis. As symptoms worsened, he returned to the ED.

On initial evaluation in our ED, he complained of persistent moderate dorsolumbar pain and productive cough, without shortness of breath. There was no history of vomiting, aspiration, or other abdominal complaints.

On physical examination, there were no signs of respiratory distress and no jaundice and, upon cardiac and pulmonary auscultation, no abnormal sounds were detected. He was hemodynamically stable, with no fever; pain was recorded as 6/10 (numeric rating scale for pain) on arrival but resolved soon after admission. Thoracic X-ray showed no pleural or lung parenchymal lesions. According to previous sputum microbiological isolate,* Escherichia coli* ESBL+, he was started on endovenous meropenem and was admitted to the general ward.

He had a metastatic colorectal cancer (CCR), diagnosed in 2013; the tumor was* RAS* mutated; the disease was staged as oligometastatic liver disease at the beginning. He was treated with primary chemotherapy, mFOLFOX6 (5-fluorouracil, leucovorin, and oxaliplatin) and bevacizumab, from November 2013 to November 2014 and had a selective right portal vein embolization in June 2014, in order to be able to perform liver surgery with adequate remnant liver. On February 2015, a sigmoid colectomy was performed as well as liver segmentectomy of the VI and VII segments, conditioned by proximity to vascular structures and cholecystectomy. Surgical resection specimen confirmed liver metastasis with complete pathological response of the primary lesion, staged as ypT0N0M1. It was considered a R1 surgery due to liver margin intersection (vascular margin).

Postoperative period was prolonged due to bilioenteric fistula and liver abscess adjacent to the segmentectomy scar that evolved to septic shock; blood cultures and peritoneal drainage were positive for* Escherichia Coli*, ESBL+.

He was discharged on the 40th postoperative day (May 2015) after successful medical treatment (large spectrum antibiotic and interventional radiology drainage), with normal liver tests and no abnormal findings on CT scan.

He had no further complementary chemotherapy.

On January 2016 lung and liver progression (relapse on the surgical scar) were detected on CT scan. Lesions were considered unresectable. Chemotherapy was restarted with mFOLFOX6 combined with bevacizumab, receiving 4 cycles until this hospital admission.

**Figure 1:**
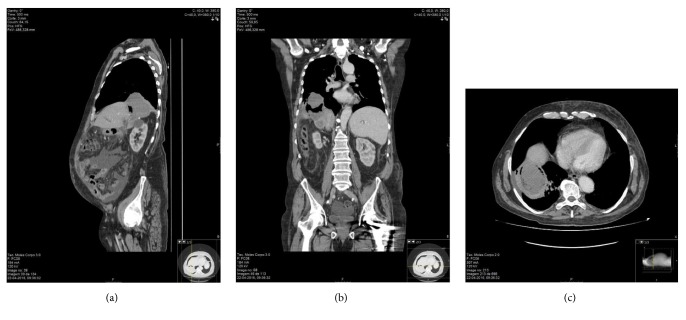
CT-Scan performed in April 2016: (a) sagittal plane, (b) coronal plane, and (c) transverse plane.

Initial cultural exams confirmed* Escherichia Coli* ESBL+ respiratory infection associated with same agent bacteremia; the antibiotic susceptibilities test was identical to the previous one; cultural exams turned negative after the first week of antibiotic. Although inflammatory parameters and arterial gasometry improved, he kept daily fever (>38°C) and expelled large volumes of yellow-green sputum (>1L per day); as symptoms kept ongoing he had a chest and abdomen CT scan on hospitalization day 15. In the liver, there was a lesion with air and necrotic component adjacent to the metastatic subdiaphragmatic lesion scar relapse and adjacent lung atelectasis (Figure 1). No fistula trajectory was evident. There was no pleural or pericardial effusion.

A bronchoscopy was performed showing right middle bronchus obstruction with a large amount of thick and brown secretions (with microbiological isolate of* Escherichia coli* ESBL+). Interventional radiology drainage for the subdiaphragmatic metastatic abscess was performed; the possibility of a fistula between the abscess and the lung was confirmed after local instillation of radiological contrast that the patient expelled with cough.

After drainage, the patient had a significant clinical improvement; 1 month later, with only residual liver drainage, the drain was removed.

While on antibiotics, the patient received inpatient rehabilitation and respiratory therapy and was successfully discharged on the 63rd day.

He was then followed-up as an outpatient in the gastrointestinal cancer clinic, with progressive resolution of the respiratory symptoms and no clinical evidence of recurrence of the fistula.

## 3. Discussion

Hepatopulmonary fistula (HPF) is a rare condition, which was classically associated with hydatic disease or hepatic amebiasis. However, since its first report in 1850, and according to a review of 68 cases between 1980 and 2010 (published in 2011), etiological primary condition has been evolving and cancer already represents the main cause (primary cancer of the liver or bile duct in 13 cases and liver metastases in 9 out of 68 cases) [5].

There are two major ways of fistula formation described in literature: mechanical (bile duct obstruction) and infectious (subphrenic or intrahepatic abscess), which cannot be completely separated. The mechanical effect of an infected biloma underneath the diaphragm is related also to tissue erosion by bile, reaching the pleural space, bronchus, or both [2, 6]. Biloma formation can be caused by diaphragmatic injuries with concomitant liver trauma, tumors (most common cause according to literature), postoperative or post ablating biliary stenosis, and lithiasis. The inflammatory process related to spreading of a hydatic liver cyst or other invasive liver processes (e.g., amoebic abscess) to the adjacent lung or pleural space is the main pathway in these conditions, usually not associated with biliary obstruction.

The mechanism reported for hepatopulmonary fistula after hepatectomy of liver metastases is through direct fistulization between biliary and bronchial tree.

In the present case, we probably have mixed mechanisms, both mechanical and inflammatory/infectious: surgery, electrical margin fulguration, chemotherapy, anti-VEGF, and infection.

After liver surgery, there was a postoperative liver abscess, which resolved with antibiotic and liver drainage. Although clinically resolved, the sequelae hepatic parenchyma changes may have contributed to the vulnerable territory for the development of fistulization.

Subsequently, there was a liver scar recurrence, and the patient started chemotherapy and bevacizumab. As the patient restarted this therapeutic, the immune system depression coupled with the antiangiogenic effect of the antibody led the way to necrosis, infection reactivation, erosion of the diaphragm, and HPF. The sputum was in fact necrotic liver and purulent material from the liver abscess; there was no confirmation of bile drainage and the fistulography did not confirm biliary involvement.

As the microbiological agent identified had the same morphological characteristics and antibiotic sensitivity as the one isolated in 2015, we assumed that the metastasectomy locus might be in fact a silent focus of infection.

We reviewed the surgery report and there was no mention of any diaphragmatic laceration that could possibly be associated with the fistula development.

According to previous reports, classical clinical presentation of HPF is irritant cough productive of copious dark-yellow sputum (bilioptysis), which is a very specific symptom of biliary bronchial fistula, with pneumonitis or bronchiolitis resulting from inflammatory reaction of the bronchial mucosa secondary to bile [7].

In the literature, endoscopic retrograde cholangiopancreatography (ERCP) and percutaneous transhepatic cholangiography are considered the methods of election for the diagnosis, although thoracic-abdominal CT scan is the most commonly used method to approach these patients [8]. Bronchofibroscopy is considered of little benefit as a diagnostic tool, except when there is a high suspicion of primary lung neoplasm.

The therapeutic approach depends primarily on the underlying pathology, but always involves invasive techniques of drainage, either percutaneously or endoscopically, eventually coupled with surgery, as conservative medical treatment has been shown to be insufficient in all reported cases [2, 5, 9, 10].

This patient presents a complication that occurs 14 months after liver surgery, and his primary symptoms are respiratory, without gastrointestinal complaints. In addition, no asymmetries of the lung fields or pleural effusion were identified in the chest radiograph, which could indicate a “localized” inflammatory/infectious process.

It is not a direct fistulization between the biliary tree and the bronchial tree, due to a stenosis or obstruction by the tumor, but an iatrogenic hepatic abscess with pulmonary drainage.

## 4. Conclusion

HPF is a rare condition, with increasing incidence secondary to liver surgery and local ablative techniques, occurring at variable time after surgery.

In patients with liver local treatments, presenting with fever, respiratory, and upper GI symptoms differential diagnosis should include HPF, as if not adequately treated it has a dismal prognosis. Imaging performing, with CT scan, EPRC, and CPT, may be of help in diagnosis if hepatobiliary fistula is suspected. Also, an accessible and noninvasive way of confirming this hypothesis would be the determination of bilirubin values in sputum analysis.

With the increasing use of interventional techniques in primary and secondary neoplasms of the liver, in view of a refractory infectious condition, especially in diabetic patients undergoing liver surgery, this disorder should be considered as a possible late complication, since it implies a very specific and directed approach.
